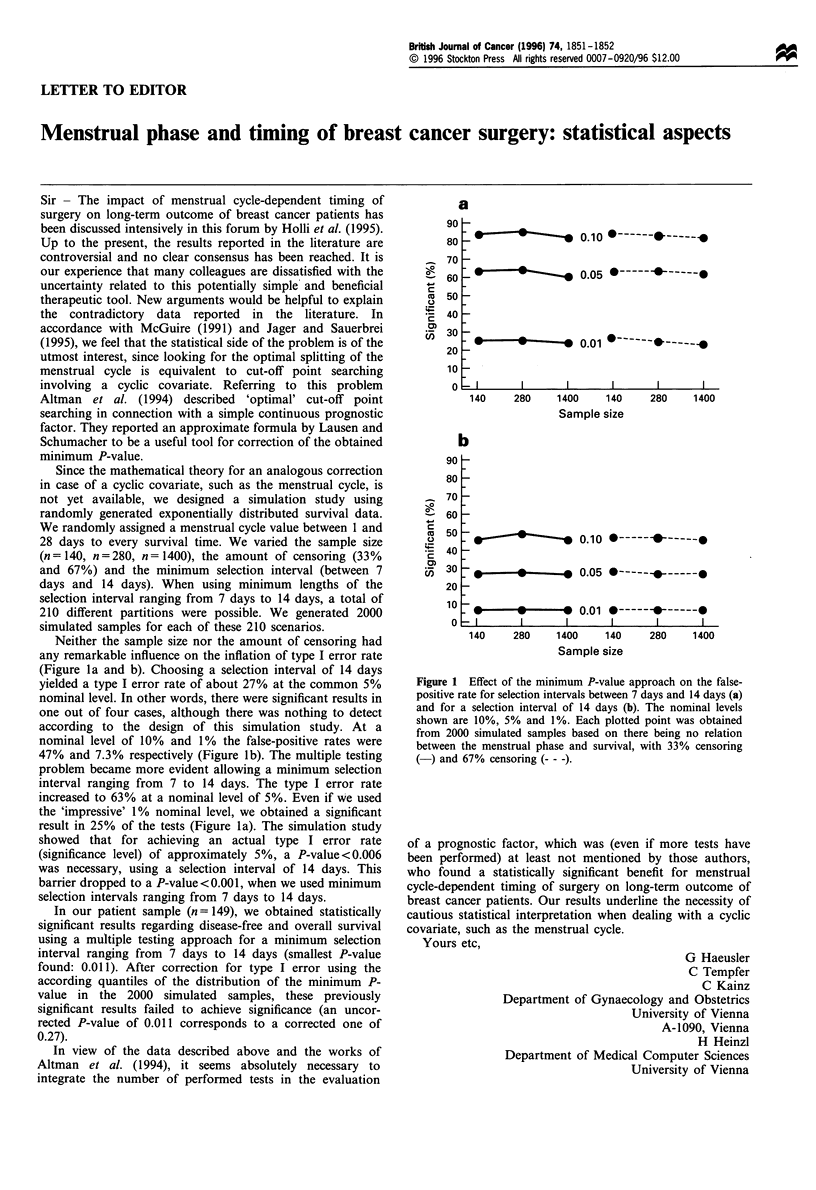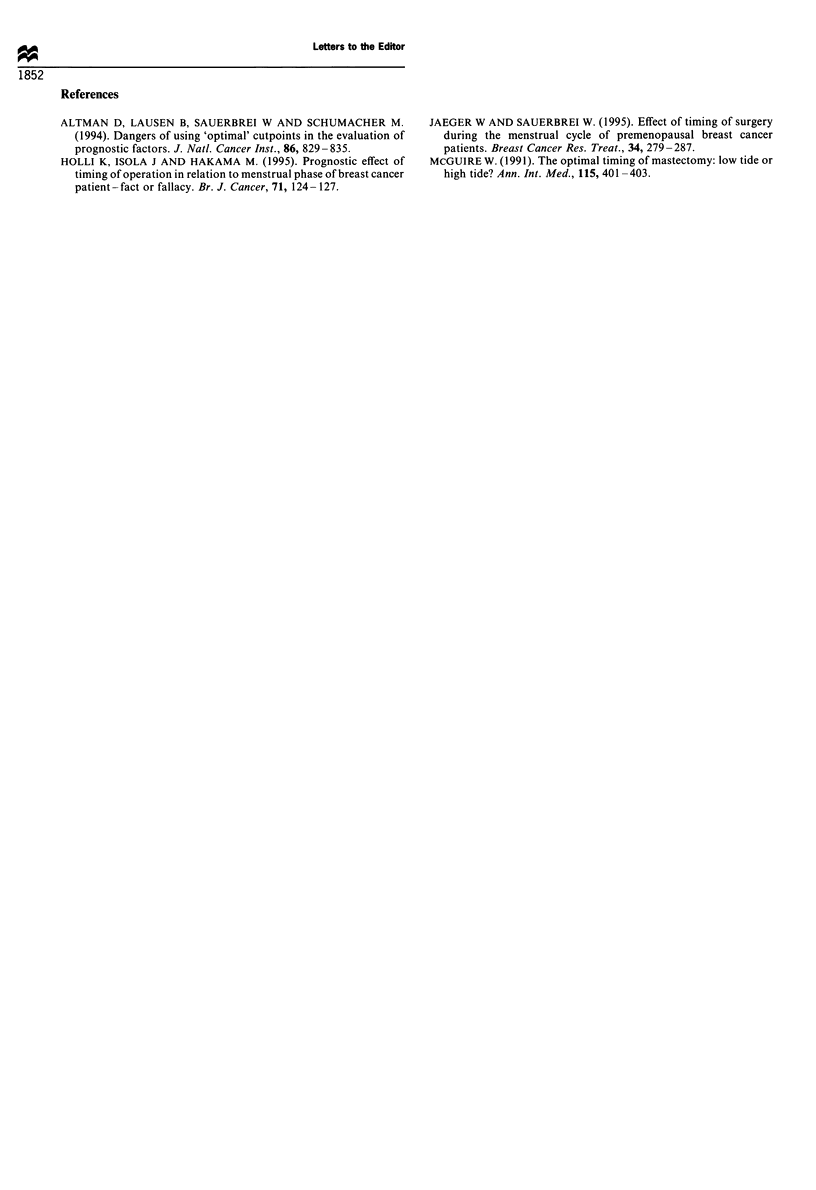# Menstrual phase and timing of breast cancer surgery: statistical aspects.

**DOI:** 10.1038/bjc.1996.643

**Published:** 1996-12

**Authors:** G. Haeusler, C. Tempfer, C. Kainz, H. Heinzl


British Journal of Cancer (1996) 74, 1851-1852
© 1996 Stockton Press All rights reserved 0007-0920/96 $12.00

LETTER TO EDITOR

### Menstrual phase and timing of breast cancer surgery: statistical aspects

Sir – The impact of menstrual cycle-dependent timing of surgery on long-term outcome of breast cancer patients has been discussed intensively in this forum by Holli *et al.* (1995). Up to the present, the results reported in the literature are controversial and no clear consensus has been reached. It is our experience that many colleagues are dissatisfied with the uncertainty related to this potentially simple and beneficial therapeutic tool. New arguments would be helpful to explain the contradictory data reported in the literature. In accordance with McGuire (1991) and Jager and Sauerbrei (1995), we feel that the statistical side of the problem is of the utmost interest, since looking for the optimal splitting of the menstrual cycle is equivalent to cut-off point searching involving a cyclic covariate. Referring to this problem Altman *et al.* (1994) described 'optimal' cut-off point searching in connection with a simple continuous prognostic factor. They reported an approximate formula by Lausen and Schumacher to be a useful tool for correction of the obtained minimum *P*-value.

Since the mathematical theory for an analogous correction in case of a cyclic covariate, such as the menstrual cycle, is not yet available, we designed a simulation study using randomly generated exponentially distributed survival data. We randomly assigned a menstrual cycle value between 1 and 28 days to every survival time. We varied the sample size ($n=140$, $n=280$, $n=1400$), the amount of censoring (33% and 67%) and the minimum selection interval (between 7 days and 14 days). When using minimum lengths of the selection interval ranging from 7 days to 14 days, a total of 210 different partitions were possible. We generated 2000 simulated samples for each of these 210 scenarios.

Neither the sample size nor the amount of censoring had any remarkable influence on the inflation of type I error rate (Figure 1a and b). Choosing a selection interval of 14 days yielded a type I error rate of about 27% at the common 5% nominal level. In other words, there were significant results in one out of four cases, although there was nothing to detect according to the design of this simulation study. At a nominal level of 10% and 1% the false-positive rates were 47% and 7.3% respectively (Figure 1b). The multiple testing problem became more evident allowing a minimum selection interval ranging from 7 to 14 days. The type I error rate increased to 63% at a nominal level of 5%. Even if we used the 'impressive' 1% nominal level, we obtained a significant result in 25% of the tests (Figure 1a). The simulation study showed that for achieving an actual type I error rate (significance level) of approximately 5%, a *P*-value<0.006 was necessary, using a selection interval of 14 days. This barrier dropped to a *P*-value<0.001, when we used minimum selection intervals ranging from 7 days to 14 days.

In our patient sample ($n=149$), we obtained statistically significant results regarding disease-free and overall survival using a multiple testing approach for a minimum selection interval ranging from 7 days to 14 days (smallest *P*-value found: 0.011). After correction for type I error using the according quantiles of the distribution of the minimum *P*-value in the 2000 simulated samples, these previously significant results failed to achieve significance (an uncorrected *P*-value of 0.011 corresponds to a corrected one of 0.27).

In view of the data described above and the works of Altman *et al.* (1994), it seems absolutely necessary to integrate the number of performed tests in the evaluation

**Figure 1** Effect of the minimum *P*-value approach on the false-positive rate for selection intervals between 7 days and 14 days (**a**) and for a selection interval of 14 days (**b**). The nominal levels shown are 10%, 5% and 1%. Each plotted point was obtained from 2000 simulated samples based on there being no relation between the menstrual phase and survival, with 33% censoring (—) and 67% censoring (- - -).

of a prognostic factor, which was (even if more tests have been performed) at least not mentioned by those authors, who found a statistically significant benefit for menstrual cycle-dependent timing of surgery on long-term outcome of breast cancer patients. Our results underline the necessity of cautious statistical interpretation when dealing with a cyclic covariate, such as the menstrual cycle.

Yours etc,

G Haeusler
C Tempfer
C Kainz
Department of Gynaecology and Obstetrics
University of Vienna
A-1090, Vienna
H Heinzl
Department of Medical Computer Sciences
University of Vienna

### References

ALTMAN D, LAUSEN B, SAUERBREI W AND SCHUMACHER M. (1994). Dangers of using 'optimal' cutpoints in the evaluation of prognostic factors. *J. Natl. Cancer Inst.*, **86**, 829–835.

HOLLI K, ISOLA J AND HAKAMA M. (1995). Prognostic effect of timing of operation in relation to menstrual phase of breast cancer patient–fact or fallacy. *Br. J. Cancer*, **71**, 124–127.

JAEGER W AND SAUERBREI W. (1995). Effect of timing of surgery during the menstrual cycle of premenopausal breast cancer patients. *Breast Cancer Res. Treat.*, **34**, 279–287.

MCGUIRE W. (1991). The optimal timing of mastectomy: low tide or high tide? *Ann. Int. Med.*, **115**, 401–403.